# A new dressing system for accelerating wound recovery after primary total knee arthroplasty: a feasibility study

**DOI:** 10.1186/s12893-024-02409-z

**Published:** 2024-04-15

**Authors:** Pengfei Lei, Fawei Gao, Jun Qi, Zhigang Li, Da Zhong, Shilong Su

**Affiliations:** 1 Department of Orthopedics, The First Affiliated Hospital, College of Medicine, Zhejiang University, No.1367 West Wenyi Road, 310003 Hangzhou, Zhejiang Province China; 2 Hunan Engineering Research Center of Biomedical Metal and Ceramic Implants, Xiangya Hospital, Central South University, No.87 Xiangya Road, 410008 Changsha, Hunan Province China; 3 Department of Orthopedics, Xiangya Hospital, Central South University, No.87 Xiangya Road, 410008 Changsha, Hunan Province China; 4 Hunan key laboratary of aging biology, Xiangya Hospital, Central South University, No.87 Xiangya Road, 410008 Changsha, Hunan Province China; 5 Department of Orthopedics, Peking University Third Hospital, No.49 North Garden Road. Haidian, 100191 Beijing, China

**Keywords:** Dressing, Dressing change, Waterproof, Knee arthroplasty, Feasibility

## Abstract

**Purpose:**

Currently, postoperative wound infection and poor healing of total knee arthroplasty have been perplexing both doctors and patients. We hereby innovatively invented a new dressing system to reduce the incidence of postoperative wound complications.

**Methods:**

We enrolled 100 patients who received primary unilateral total knee arthroplasty and then applied the new dressing system. The data collected included the number of dressing changes, postoperative hospital stay, Visual Analogue Scale score (VAS), the Knee Society Score (KSS), the Knee Injury and Osteoarthritis Outcome Score (KOOS), ASEPSIS scores, The Stony Brook Scar Evaluation Scale (SBSES), wound complications, dressing cost, the frequency of shower and satisfaction. Subsequently, a statistical analysis of the data was performed.

**Results:**

Our findings demonstrated the average number of postoperative dressing changes was 1.09 ± 0.38, and the average postoperative hospital stay was 3.72 ± 0.98 days. The average cost throughout a treatment cycle was 68.97 ± 12.54 US dollars. Collectively, the results of VAS, KSS, and KOOS revealed that the pain and function of patients were continuously improved. The results of the four indexes of the ASEPSIS score were 0, whereas the SBSES score was 3.58 ± 0.52 and 4.69 ± 0.46 at two weeks and one month after the operation, respectively. We observed no wound complications until one month after the operation. Remarkably, the satisfaction rate of the patients was 91.85 ± 4.99% one month after the operation.

**Conclusion:**

In this study, we invented a new dressing system for surgical wounds after total knee arthroplasty and further confirmed its clinical feasibility and safety.

**Chinese clinical trial registry:**

ChiCTR2000033814, Registered 13/ June/2020.

## Introduction

In the process of clinical diagnosis and treatment of osteoarthritis and severe rheumatoid arthritis, artificial joint arthroplasty is the main treatment strategy, which can relieve the pain of patients [1, 2]. With the aging of the population, the number of joint arthroplasties has been increasing substantially. It has been reported that the recovery management of patients after joint arthroplasty is critical, and one of the most important steps in the management is surgical wound [3]. Wound healing is a complex process that is associated with the growth and regeneration of cells and tissues, which is affected by internal and external factors [4]. It has been demonstrated that once postoperative complications such as poor wound healing occur, it is very likely to lead to a more terrifying complication of the joint arthroplasty, for instance, periprosthetic joint infection. The occurrence of periprosthetic joint infection will increase the recovery time, hospitalization days, medical expenses, and pain of patients, causing a huge burden to the family and society.

Previous reports have estimated that the proportion of total knee arthroplasty (TKA) patients undergoing revision due to postoperative infection is around 29%, which is one of the main reasons for TKA revision [5]. Therefore, wound dressings and some special equipment that can help and facilitate the process of wound healing form an important part of wound management. In particular, wound dressings are designed to establish and maintain optimal conditions to reconstruct the damaged or lost tissues. The ideal properties of wound dressings include the provision of a moist environment to avoid wound dryness, prevention of any contamination, absorption of wound exudates, stimulation of cell growth factors, adequate mechanical strength and flexibility, and biocompatibility/biodegradability [6]. However, in our medical institutions, the standard wound dressing is still the traditional gauze dressing, which lacks most of the above-mentioned characteristics. Most patients often complain about pain during dressing change and discomfort during knee exercise when using gauze dressing after the operation, especially when the range of motion is more than 45 degrees. Notably, skin blisters caused by movement around the joint leading to friction between the skin and traditional gauze dressing has been associated with postoperative infection [7]. In this respect, traditional gauze dressing does not meet the ideal dressing requirements of TKA. Thus, there is an urgent for the development of a new dressing system.

Calcium alginate dressing [8], a new type of dressing, is characterized by rapid and strong absorption capacity of exudate, can absorb liquid-equivalent up to 17–20 times of its weight, can effectively control exudation, and prolong the time of dressing change [9]. As a result, keep the wound moist without adhering to it, protect exposed nerve endings, promote wound healing, and relieve pain. It also has a hemostatic function [10], making it a new dressing with good application prospects. However, at the present stage, calcium alginate dressings are often used in combination with gauze dressings, which makes them unable to overcome the shortcomings of gauze dressings, as well as limit the benefits of calcium alginate dressings, such as prolonging the time of dressing change. To solve this clinical problem, we creatively applied IV3000 film and calcium alginate dressing to the surgical wound management of patients undergoing TKA. IV3000 film refers to a kind of dressing film for intravenous catheterization, which has high moisture permeability [11], good waterproof performance, inhibition of bacterial colonization [12], less friction with skin, and almost no pain during removal [13]. Of note, this dressing makes use of both the benefits of calcium alginate dressing, as well as the characteristics of IV3000, hence inventing a new dressing system.

On this background, we herein designed this clinical trial to confirm the clinical feasibility and safety of this new dressing system. We performed this clinical trial by recording the number of postoperative dressing changes, postoperative hospitalization days, dressing cost, wound complications, functional recovery and quality of life of patients, the frequency of shower and self-evaluation of satisfaction.

## Patients and methods

The protocol of this study was approved by the medical ethics committee of our hospital, and written informed consent was obtained from all enrolled patients. This study was registered at www.chictr.org.cn (ChiCTR2000033814).

Patient inclusion criteria were as follows: (1) age 18 to 85 years; (2) according to the physical examination and the history of osteoarthritis confirmed by clinical and radiology data; and (3) planning to undergo primary unilateral TKA. On the other hand, exclusion criteria included: (1) undergone open knee surgery on any knee joint; (2) having deformities caused by severe knee trauma; (3) having clear scars in any knee joint; (4) being allergic to skin adhesion; (5) suffering from skin diseases such as psoriasis, eczema, or dermatitis; and (6) being unable to finish the regular follow-up. Sample size calculation: as per the previous study [14] and our preliminary results, we set alpha = 0.05, beta = 0.1, the mean difference of dressing change was 1.0 compared to traditional gauze dressing. A minimum of 50 patients would be needed to provide 90% power.

In this study, we enrolled a total of 100 patients between August 1, 2020, and December 23, 2021. Specifically, there were 27 males and 73 females, with a median age of 66.56 ± 7.41 years (range 51–82 years), 47 on the left, and 53 on the right. The demographic characteristics were shown in Table 1. All TKAs were conducted by an experienced joint surgeon. In all patients, a standard midline skin incision with a medial parapatellar capsulotomy was performed. All prostheses were fixed with bone cement. Prophylactic antibiotic cefoxitin was routinely administered in preoperative 30 min.

**Table 1:** Demographic characteristics of the patients

Characteristics	Subjects (*n* = 100)
Age, year	
Median age	66.56 ± 7.41
Range	51–82
Age distribution, year, No (%)	
50–60	20 (20%)
60–70	52 (52%)
70–85	28 (28%)
Gender	
Males	27 (27%)
Females	73 (73%)

### Application of new dressing system

**Fig. 1:**
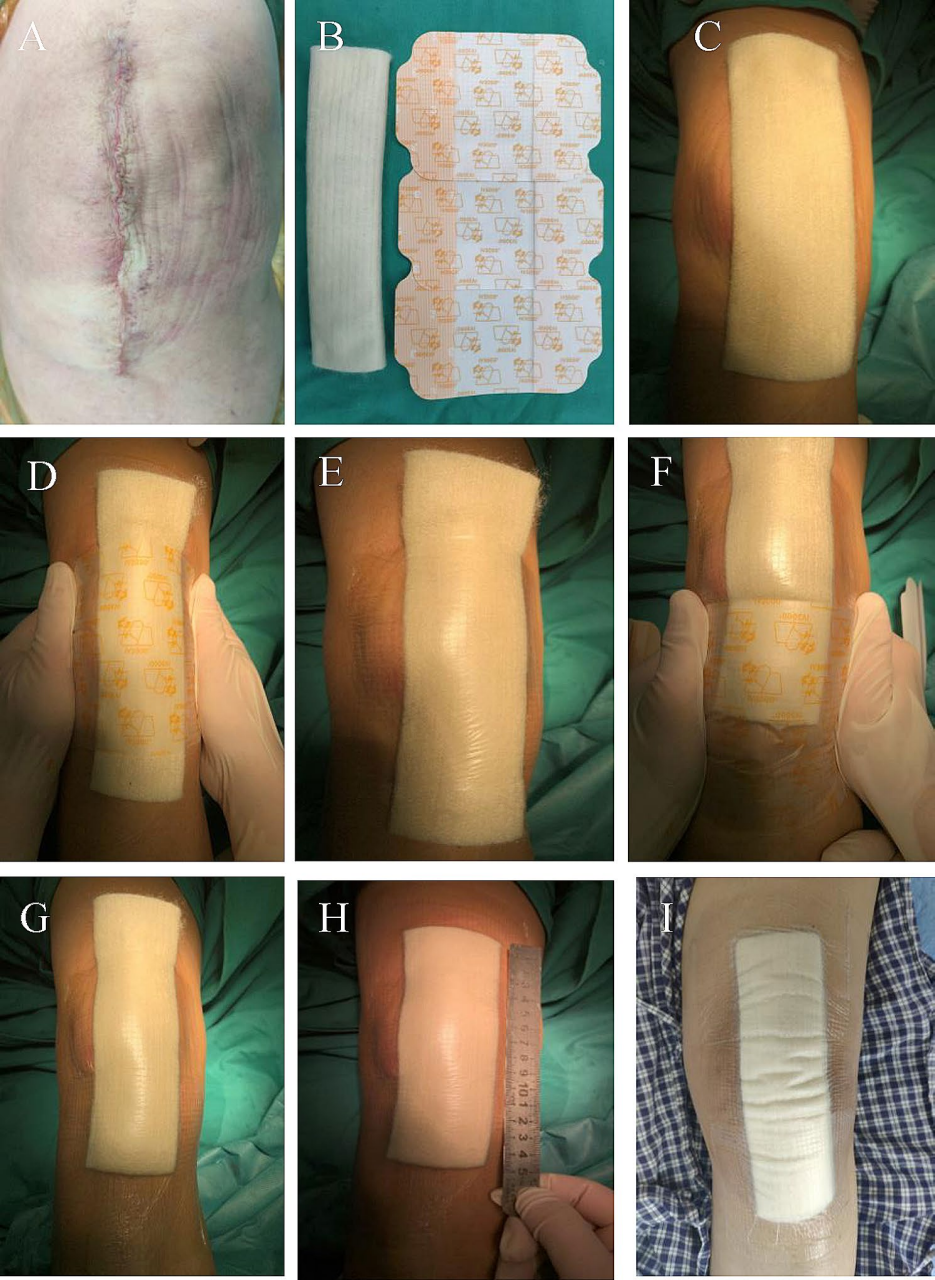
**A**. The wound was sutured and deiodized. **B.** Folded calcium alginate dressing and 3 IV3000 films. **C.** Cut the calcium alginate dressing to both ends slightly longer than the incision 1 cm. **D-H.** The middle one is pasted first, and then the film is pasted from the distal end to the proximal end. The two ends of the films were slightly longer than the incision about 4 cm, and the overlap between the two films was about 1 cm. There is no air bubble between the films and the skin and stick to the skin tightly. **I.** After the patient took a bath according to his own habits, the dressing was not affected

The application of the new dressing system has been described in detail in our previous study [14]. In brief, after the suture of the surgical incision, fold the calcium alginate dressing (Algisite M, Smith &Nephew, London, UK) into three layers in the long axis direction and place it on the incision; when the knee flexion was about 45 degrees, 3 to 4 pieces of IV3000 film (Smith &Nephew, London, UK) were selected and pasted. There was no air bubble between the films and the skin, thus sticking to the skin tightly (Fig. 1).

All patients adopted the same nursing measures after the operation. On the first day after the operation, the ice elastic bandage was removed, followed by 3 days of treatment with prophylactic antibiotic cefoxitin, and then enoxaparin sodium 4000IU was subcutaneously injected to prevent deep venous thrombosis. During the hospital stay, patients with the new dressing system were not required to change their dressings if there was no obvious large amount of exudation, scratches, or crimps, but only once on the day of discharge. Additionally, there was no need to change them after discharge. After the operation, patients were free to take a normal shower based on their living habits (Fig. 1).

### Data collection

In general, the data collected in this study comprised the number of dressing changes, postoperative hospital stay, dressing cost, pain and function scores, wound scores and complications, shower frequency and satisfaction, as described in the following subsections.

### Number of dressing changes, postoperative hospital stay and dressing cost

When patients meet strict standards, such as the ability to perform independent personal care, walk at least 70 m on crutches, get in and out of bed and get up from chairs, and managed with oral pain relief [15], they were discharged from the hospital. The postoperative hospital stay was computed as the whole day, whereas the part less than one day was also calculated as one day. After discharge, the patient was assigned to a chat group, whereby the dressing was photographed and evaluated under the guidance of medical staff. All patients were not covered with dressing two weeks after the operation. Afterward, we recorded the total number of dressing changes. For the sake of safety, we changed the dressing and observe the wound on the day the patient was discharged from the hospital. This might have led to an increase in the total number of dressing changes, but we thought it was necessary. Lastly, we recorded the medical expenses incurred by patients using the new dressing in order to establish its average cost throughout the treatment cycle.

### Pain and function scores

To record the pain, and function of patients, as well as to assess the changes of perioperative patients, we used the Visual Analogue Scale score (VAS) [16], the Knee Society Score (KSS) [17, 18], and the Knee injury and Osteoarthritis Outcome Score (KOOS) [19]. The time points of the evaluation were recorded within one week before the operation, and on the day one month after the operation.

### Wound scores and complications

The ASEPSIS score is a commonly used wound assessment score [20, 21]. In this work, we only used the objective wound assessment section of this score [22], because we only aimed to determine the clinical appearance of the wound. Moreover, the Stony Brook Scar Evaluation Scale (SBSES), proposed by Singer et al. in 2007 [23], is a wound evaluation scale used to measure the cosmetic effect of a wound, including the width, height, color, residual suture marks, and an overall view of the scar. The score of each index is either 0 or 1, and the total score is calculated, ranging from 0 (worst) to 5 (best). The ASEPSIS and SBSES scores were recorded at two weeks and one month after the operation. The special follow-up technician was based on where the photos were taken or on-site observation records. Simultaneously, the wound complications of patients during each follow-up period were recorded and photographed within one month after the operation.

### Shower frequency and satisfaction

In this subsection, we developed a questionnaire to conduct the shower frequency and satisfaction survey. The questionnaire recorded patients’ satisfaction with eight parameters, including their comfort with dressings, ability to take a shower, pain treatment, doctor visits, length of stay, number of dressing changes, hospitalization costs, and satisfaction with the overall experience. All measurements were recorded in numerical terms, with a score of 0 to 10, with a maximum score of 80. Then, one month after the operation, the patients filled in the records according to their real situation.

All data were collected by an independent researcher who was not involved in experimental design and surgery. All quantitative data were expressed as mean ± standard deviation. The normal distribution test (Kolmogorov-Smirnov test) and homogeneity test (Levene test) were first performed. If the data conformed to the normal distribution and homogeneity, the paired t-test was used. If not, the non-parametric test was used. A value of *P* < 0.05 was considered statistically significant. Data analysis was executed using SPSS software, version 25.0 (SPSS, USA).

## Result

### Number of dressing changes, postoperative hospital stay and dressing cost

We found that the average times of dressing change were 1.09 ± 0.38, and the average postoperative hospital stay was 3.72 ± 0.98 days. We also uncovered that the application of the new dressing system required an average of 1 calcium alginate dressing and 3 IV3000 films. Notably, the cost of one dressing change was 33 US dollars. The average cost of the new dressings throughout a treatment cycle was 68.97 ± 12.54 US dollars.

### Pain and function scores

Here, we employed VAS, KSS, and KOOS scores to measure the pain, and function of the patients within one week before the operation, and on the day one month after the operation. The VAS score decreased from 5.75 ± 1.39 one week before the operation to 0.73 ± 0.72 one month after the operation. On the other hand, the knee and function score of the KSS increased from 55.69 ± 19.32 and 52.89 ± 22.91 before the operation to 87.65 ± 4.73 and 73.60 ± 8.99 one month after the operation, respectively. We also identified that the KOOS of pain, symptoms, ADL, function in sports and recreation, and QOL were significantly improved one month after the operation compared with one week before the operation (Table 2).

**Table 2:** The score results of VAS, KSS, and KOOS

Variable	Preoperative	one months postoperatively	t value	*P* value
VAS	5.75 ± 1.39	0.73 ± 0.72	32.244	< 0.001
KSS				
Knee	55.69 ± 19.32	87.65 ± 4.73	15.767	< 0.001
Function	52.89 ± 22.91	73.60 ± 8.99	9.006	< 0.001
KOOS				
Pain	50.00 ± 20.78	84.64 ± 9.68	16.535	< 0.001
Symptoms	45.00 ± 16.89	88.25 ± 6.52	22.373	< 0.001
ADL	39.89 ± 14.38	91.24 ± 4.97	33.995	< 0.001
Function in sports and recreation	22.95 ± 22.66	72.85 ± 11.04	20.579	< 0.001
QOL	32.38 ± 20.14	63.81 ± 11.11	13.608	< 0.001

### Wound scores and complications

**Fig. 2:**
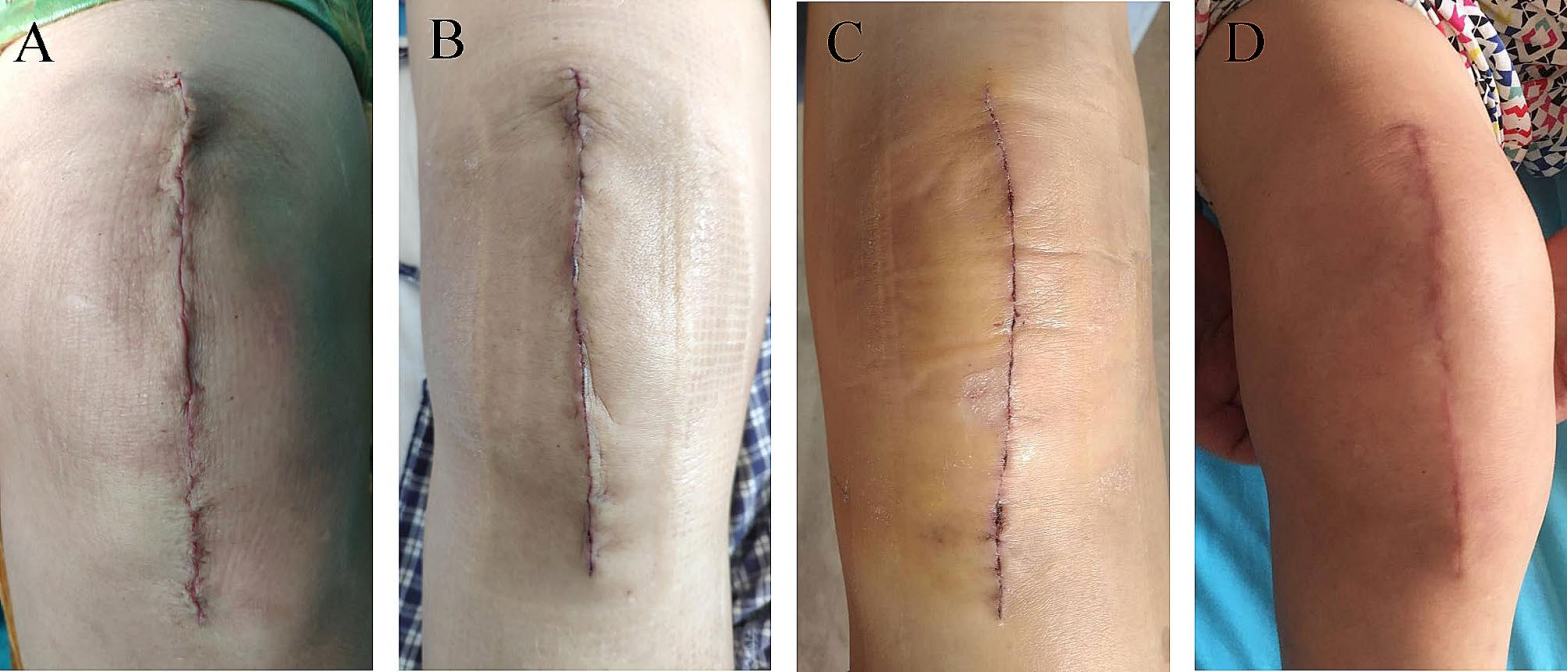
**A**. Shows wound sutured during the operation. **B.** There was no obvious ecchymosis, swelling, and exudation in the wound three days after the operation. **C.** The wound healed completely two weeks after the operation. **D.** One month after the operation, the wound of the patient showed that the scar was smooth, consistent with the color of the surrounding skin, and the overall appearance was satisfactory

The findings of serious discharge, erythema, purulent discharge, and wound defect based on the ASEPSIS score were all 0 (Table 3). While the SBSES scores were 3.58 ± 0.52 and 4.69 ± 0.46 at two weeks and one month after the operation, respectively (Table 3). We correspondingly found that the appearance of the wound improved gradually with the prolongation of recovery time. Noticeably, there was no wound complication recorded until one month after the operation. Interestingly, the wound of patients healed well, hence classifying their scars as comfortable and satisfactory in appearance (Fig. 2).

**Table 3:** The score results of the ASEPSIS and SBSES

	Two weeks postoperatively	One month postoperatively	t value	*P* value
ASEPSIS				
Serous discharge	0	0		1.000
Erythema	0	0		1.000
Purulent discharge	0	0		1.000
Wound defect	0	0		1.000
SBSES				
Width	0.89 ± 0.31	0.94 ± 0.24	1.216	0.227
Height	0.88 ± 0.33	0.96 ± 0.20	2.031	0.045
Color	0.00 ± 0.00	0.96 ± 0.19	48.744	< 0.001
Residual suture marks	1.00 ± 0.00	1.00 ± 0.00		1.00
The overall view	0.95 ± 0.22	1.00 ± 0.00	1.421	0.185
Total score	3.58 ± 0.52	4.69 ± 0.46	2.283	0.025

SBSES: Stony Brook Scar Evaluation Scale

### Shower frequency and satisfaction

The patient’s shower frequency was shown in Table 4 and 8 patients (8%) did not take showers because they were afraid of getting wound infections. Most patients (78/100, 78%) took showers once per day. One month after the operation, we found that the satisfaction of the patients were 73.34 ± 3.86, and the satisfaction rate was 91.85 ± 4.99%.

**Table 4:** The results of the shower frequency

Frequency	Number	n%
No shower	8	8%
Twice per day	5	5%
Once per day	78	78%
Every 2 days	7	7%
Every 3 days	2	2%

## Discussion

In this present investigation, we have invented a new dressing system by combining IV3000 film and calcium alginate dressing in a specific way for surgical wounds after TKA. Subsequently, this prospective single-arm clinical study confirmed the clinical feasibility and safety of the new dressing system and also verified many benefits over the commonly used clinical gauze dressings, such as flexibility, and water-resistant, among others.

With the recent development of the field of knee arthroplasty and the continuous promotion of the concept of rapid rehabilitation, patients are encouraged to get out of bed early for exercise and early discharge for rehabilitation [24]. The rapid rehabilitation of patients with TKA needs a comprehensive development of many areas to better achieve. There are myriad problems involved in wound management, but there is huge room for improvement. Presently, in the Chinese hospital, most of the wounds after TKA are still covered with traditional gauze dressings. Due to the high range of motion of the knee joint, traditional gauze dressings are only fixed with adhesive tape, which is easy to fall off, thus surgical wound is not effectively protected and the frequency of dressing change is increased. In general, most patients in China choose to go home directly to recuperate and lack the concept of entering rehabilitation hospitals for rehabilitation. In this way, there is an increased risk of dressing changes without professional guidance in wound management at home after the operation. Each patient is required to prepare the skin regularly and take a bath on the day before the operation, which is expected to reduce the risk of bacterial infection on the skin around the surgical incision. Additionally, washing the skin after surgery is crucial, but wounds covered with traditional gauze dressings cannot be wet because they are not waterproof. In response to this, the Chinese patients are not to take a bath or only take a body scrub within two weeks to avoid the skin around the wound to get wet. As a result of this treatment, the skin around the wound cannot be therefore cleaned properly after the operation, significantly increasing the risk of wound infection and eventually reducing the quality of life [25].

Currently, the new method of wound dressing management can use smart dressings, especially Aquacel Ag Hydrofiber, which has been previously proved to be effective in promoting wound healing, preventing bacterial colonization infection, and reducing wound drainage [26]. However, these dressings techniques are generally expensive, hindering their wide application, particularly in developing countries and some low-income patients in rural areas. On the other hand, calcium alginate dressings exhibit more prospects for popularization and application. Unfortunately, at this stage, calcium alginate dressings are often used along with gauze dressings, which makes them unable to overcome the shortcomings of gauze dressings as well as limit the advantages of calcium alginate dressings, for example, prolonging the time of dressing change. It is important to note that the new dressing system not only gives full play to the advantages of calcium alginate dressing, such as fast and strong absorption capacity of exudate and prolonging dressing time but also increases the benefits of waterproof, and limited elasticity. In comparison, we found that the cost of dressing change was 33 US dollars, which is relatively cheaper compared to other new dressings. The average cost of the incision negative pressure wound therapy dressings in the 7-day treatment cycle was about 158.51 US dollars (125 pounds in the original article) [27]. The silver-impregnated occlusive dressings can cost up to 38.05 US dollars for a single change [28]. However, compared with the traditional gauze dressing system, the price of one dressing change is more expensive, but from the whole postoperative wound management cycle, the significant reduction in the number of dressing changes will not bring additional costs to patients.

The average number of dressing changes in the incision negative pressure wound therapy dressings group was 2.5 [27] and that in the silver-impregnated occlusive dressings group was 1.3 [29]. The average frequency of postoperative dressing change in our new dressing system was only 1.0, therefore reducing the risk of wound infection caused by frequent dressing change. This also avoids the risk of poor wound management consciousness and non-standard dressing change. Based on the properties of IV3000 film and calcium alginate dressings such as good skin adhesion, elasticity, breathable and waterproof, postoperative patients can take a bath normally without worrying about the wetting of wound dressings. Besides, they can also clean the skin around the wound, reducing bacterial colonization, which greatly reduces the risk of wound infection, as well as improve the quality of life and satisfaction of patients after the operation. The prerequisites for the discharge of patients with TKA include satisfactory surgical wounds, no complications, and satisfactory mobility [30]. The bloated and inelastic traditional gauze dressings limit the range of motion of the knee joint to a great extent, and may also cause skin blisters and hinder rehabilitation activities [31]. On the other hand, this new dressing system fits well with the skin, and the limited elasticity with variable movement overcomes the above-mentioned problems, which increases the comfort of patients, and improves the quality of life and recovery after the operation. The studies [27, 29] reported that the average postoperative hospital stay was 3.8 days for the incision negative pressure wound therapy dressings, 6.3 days for the silver-impregnated occlusive dressings. The average postoperative hospital stay of our new dressing system was 3.7 days, which significantly shortening the postoperative hospital stay.

To evaluate wound healing and possible wound complications in each period, we used the objective wound assessment section of the ASEPSIS score. The results showed that there were no wound complications one month after the operation, and the wound healed well as per the objective score of the wound. Using the SBSES score to assess the appearance of the wound, we observed that the appearance of the wound gradually improved with the extension of recovery time. The patient reported that the appearance of the wound scar was satisfactory. Based on the findings from this study, we can confirm the safety of the new dressing system in wound management of TKA. We further found that the clinical appearance of the wound exhibited some advantages.

Through the results, this trial confirmed the clinical feasibility and safety of the new dressing system, and preliminarily uncovered many benefits over the commonly used clinical gauze dressings, such as beauty, flexibility, and waterproof. To verify the advantages and disadvantages of the new dressing system compared with traditional gauze dressings, future studies incorporating larger sample sizes of clinical randomized controlled trials are needed. Of note, such clinical randomized controlled trials are already underway, and our results will be published shortly.

Of course, this study also has limitations, first of all, because this was a feasibility study, we did not set up a control group. Although it was sufficient to confirm the effectiveness and safety of the new dressing system, a control group or even a randomized controlled trial was needed to better clarify its advantages and disadvantages; furthermore, as the cost of dressings varies by region and institution, the cost assessment in this study may not be applicable to other institutions; finally, this was a single-center study with a small sample size. it also needs to be further verified by multicenter large sample studies.

## Conclusion

In this study, we invented a new dressing system for surgical wounds after total knee arthroplasty and further confirmed its clinical feasibility and safety.

## Data Availability

The datasets used and/or analysed during the current study are available from the corresponding author on reasonable request.
